# Dental implant failure and retrieval techniques; a scoping review

**DOI:** 10.3389/froh.2025.1667808

**Published:** 2025-11-26

**Authors:** Vidya K. Shenoy, Shobha J. Rodrigues, Sandipan Mukherjee, Arvind Ramanathan

**Affiliations:** 1 Department of Prosthodontics, A J Institute of Dental Sciences, Mangalore, India; 2 Department of Prosthodontics, Manipal College of Dental Sciences Mangalore, Manipal Academy of Higher Education, Manipal, India; 3 Department of Oral and Maxillofacial Surgery, Manipal College of Dental Sciences Mangalore, Manipal Academy of Higher Education, Manipal, India

**Keywords:** dental implants, implant failure, implant retrieval, explantation techniques, peri-Implantitis, trephine bur, scoping review, dental implant

## Abstract

**Background:**

Dental implants are widely used, yet failures occur and the literature on their etiology and retrieval is discrete. A consolidated map of evidence from the past decade can provide valuable guidance to clinicians and researchers.

**Objectives:**

To (1) chart biological, mechanical and patient-related factors associated with implant failure (2) catalogue techniques described for implant retrieval/explantation. (3) To illustrate publication trends in the field (1983–2025).

**Methods:**

A scoping review was conducted following PRISMA-ScR guidelines. Search strategies based on relevant keywords and MeSH terms were performed across PubMed, Scopus, and Web of Science for studies published from 1983 to June 2025. Two investigators (V. S. and S.R.) independently performed screening of the literature electronically in three databases. Clinical studies, case reports, reviews and guidelines discussing dental implant failure classification, risk factors, and explantation techniques were included in the study.

After duplicate removal and title/abstract screening, 388 records were included (human studies reporting on failed endosseous dental implants or explantation techniques). Data were charted in duplicate and synthesised descriptively; no critical appraisal or meta-analysis was performed.

**Results:**

All studies included were published between 1983 and 2025, in English language. An analysis of the included literature demonstrated a progressive rise in publications from 1983 to 2025 with a sharp increase in publications after 2015, reflecting growing clinical and research interest. Observational designs predominated (68%), followed by narrative reviews (20%) and systematic reviews/meta-analyses (8%). These studies highlighted multifactorial causes of implant failure categorized as early (0.5%–5.2%) and late failures (0.5%–7.8%). Early failures were predominantly linked to smoking, uncontrolled diabetes mellitus, poor bone quality, periodontitis, radiotherapy, titanium hypersensitivity, and surgical errors. Late failures were associated with biomechanical overload, peri-implantitis, malpositioning, and systemic medication effects Commonly reported causes of failure included peri-implantitis (≈150 studies), systemic conditions such as diabetes and osteoporosis (≈60), medication exposure (e.g., bisphosphonates, SSRIs; 24), and mechanical or prosthetic factors (≈40). Fourteen studies described implant retrieval techniques: trephine burs (7), reverse-torque devices (3), ultrasonic/piezoelectric methods (2), laser-assisted removal (1), and electrosurgery-induced thermoexplantation (1). Success rates for atraumatic retrieval ranged from 70% to 100%.

**Conclusions:**

This scoping review concluded that dental implant failure remains a complex and multifactorial challenge.

Peri-implant disease, systemic health factors, and mechanical overload are the most frequently implicated causes of implant failure. Understanding risk factors and applying evidence-based retrieval strategies enhance clinical outcomes and optimize subsequent rehabilitation options.

Trephines are the most commonly reported retrieval method, but newer minimally invasive techniques are gaining interest. Further prospective studies and standardized failure definitions are recommended.

## 1 Introduction

Dental implants have revolutionized the rehabilitation of partially and completely edentulous patients, offering superior functional and esthetic outcomes compared to conventional prostheses. Over the past two decades, their widespread use has led to steadily increasing long-term survival rates, often exceeding 95% over five years. Despite these high success rates, implant failures continue to pose a significant clinical challenge, particularly when considering biological, mechanical, and patient-related factors that can compromise osseointegration or lead to eventual loss of the implant (1–3).

Implant failure is commonly classified as either early (failure to achieve osseointegration) or late (loss of osseointegration after functional loading). Both types are multifactorial in nature and can result from peri-implant disease, biomechanical complications, surgical technique errors, poor bone quality, systemic health issues, and patient behaviors such as smoking or poor oral hygiene (4). Furthermore, the advent of newer medications—such as bisphosphonates, selective serotonin reuptake inhibitors (SSRIs), and proton pump inhibitors—has drawn attention to pharmacologic factors that may interfere with bone remodeling and implant stability.

When failure occurs, the removal of implants becomes necessary. Atraumatic retrieval is crucial to minimize damage to surrounding bone and facilitate future rehabilitation. Various techniques have been developed, ranging from conventional trephine burs to newer, minimally invasive methods such as reverse-torque devices, piezosurgery, and laser-assisted removal. However, these techniques are inconsistently reported in the literature, and their comparative effectiveness is poorly understood.

Given the growing number of implant placements globally, and the increasing diversity of patient presentations, there is a need to systematically map the evidence related to implant failure and retrieval. While multiple narrative and systematic reviews exist on specific aspects such as peri-implantitis or mechanical complications, there is no comprehensive synthesis capturing the full spectrum of causes and retrieval strategies in one framework.

Therefore, this scoping review was undertaken with two primary objectives:

1. To chart and synthesize the biological, mechanical, and patient-related factors reported in the literature as contributing to dental implant failure; and
2. To identify and categorize the various techniques described for the retrieval or explantation of failed implants, including their indications and reported outcomes.
3. To illustrate publication trends in the field (1983–2025).

This review adheres to the PRISMA-ScR (Preferred Reporting Items for Systematic Reviews and Meta-Analyses extension for Scoping Reviews) guidelines and presents an evidence-based overview to support clinical decision-making and guide future research in the field of implant dentistry.

## 2 Materials & methods

### 2.1 Study design

This scoping review was conducted following the methodological framework outlined by the Preferred Reporting Items for Systematic Reviews and Meta-Analyses extension for Scoping Reviews (PRISMA-ScR). The aim was to systematically chart the literature on (1) factors contributing to dental implant failure and (2) techniques used for implant retrieval or explantation. The review did not include quality assessment or meta-analysis, as the objective was to map the extent, range, and nature of the available evidence.

The Search was done using the following PCC framework.

Population (P): Studies which reported patients with **dental implants**, including those experiencing **implant failure** for any cause (e.g., peri-implantitis, overload, systemic conditions, medication effects)

Concept (C): **Implant failure characteristics** and the **techniques used for explantation or retrieval** of failed dental implants (e.g., trephine burs, reverse torque, piezo, laser, electrochemical)

Context (C): All **study designs** (e.g., observational studies, clinical trials, case reports, reviews) published in English from **1983 to 2025**, across all care settings and geographical regions

### 2.2 Eligibility criteria

#### 2.2.1 Inclusion criteria

• Studies involving human participants with failed **endosseous dental implants**
• Articles describing biological, mechanical, systemic, surgical or patient-related causes of implant failure
• Studies describing techniques for implant retrieval or explantation
• All study designs (e.g., observational studies, clinical trials, case reports, reviews)
• Publications between **1983 and June 2025**
• Articles with English-language titles and abstracts

#### 2.2.2 Exclusion criteria

• *in vitro* studies, animal studies, and finite element analysis
• Articles focusing solely on prosthetic complications without implant failure
• Conference abstracts or editorial pieces lacking substantive data

## 3 Information sources and search strategy

A comprehensive literature search was conducted in PubMed, Scopus, and Web of Science for articles published from inception to June 30, 2025, accessed on 30 June 2025. The search strategy included combinations of keywords and MeSH terms related to dental implants, implant failure, retrieval, removal, explantation, survival, and peri-implantitis.

The keywords and search strategy performed in various databases are summarized in Table 1.

**Table 1:** Summary of database search strategies.

Database	Search strategy
PubMed	(((((“dental implant failure"[tiab]) OR (“dental implant failure"[tiab])) OR (“dental implant loss"[tiab])) OR (“dental implant removal"[tiab])) OR (“dental implant retrieval"[tiab])) OR (“dental implant retrieval techniques"[tiab])
Scopus	(((((TITLE-ABS(”dental implant failure”)) OR (TITLE-ABS(”dental implant failure”))) OR (TITLE-ABS(”dental implant loss”))) OR (TITLE-ABS(”dental implant removal”))) OR (TITLE-ABS(”dental implant retrieval”))) OR (TITLE-ABS(”dental implant retrieval techniques”))
Web of Science	(((((“dental implant failure”) OR (“dental implant failure”) OR (“dental implant loss”)) OR (“dental implant removal”)) OR (“dental implant retrieval”)) OR (“dental implant retrieval techniques”)

## 4 Selection of sources of evidence

All search results were exported to Rayyan® and screened for eligibility by two independent reviewers (V.S. and S. R.) to maintain consistency of the reviewing process. After the removal of duplicates (*n* = 602), the remaining records (*n* = 412) underwent title and abstract screening. Full-text screening was not required for this dataset because all included records were retrieved with detailed metadata and curated prior to RIS export. Disagreements were resolved by discussion and consensus between the reviewers. The final number of studies included in the review was 388.

## 5 Data charting process

Data from each included study were extracted independently by two reviewers using a pre-piloted data charting form developed by the authors to determine the variables to be extracted. The following variables were collected:

• Authors
• Year of publication
• Study design
• Reported causes of implant failure (biological, mechanical, systemic, behavioral)
• Described retrieval/explantation technique (if applicable)
• Reported outcomes or success rates of retrieval

## 6 Synthesis of results

The extracted data were synthesized narratively and presented using descriptive statistics (e.g., frequencies, percentages). Thematic categorization was applied to group failure factors and retrieval techniques. No quantitative synthesis or critical appraisal of methodological quality was undertaken.

## 7 PRISMA flow diagram summary

The selection process is summarised as shown in Table 2.

**Table 2:** Summary of study selection Process (PRISMA-ScR).

Stage	Count
Records identified from databases	1,014
Duplicates removed	602
Records screened (title/abstract)	412
Records excluded	24
Studies included for data extraction	**388**

## 8 Results

### 8.1 Study selection

**Figure 1:**
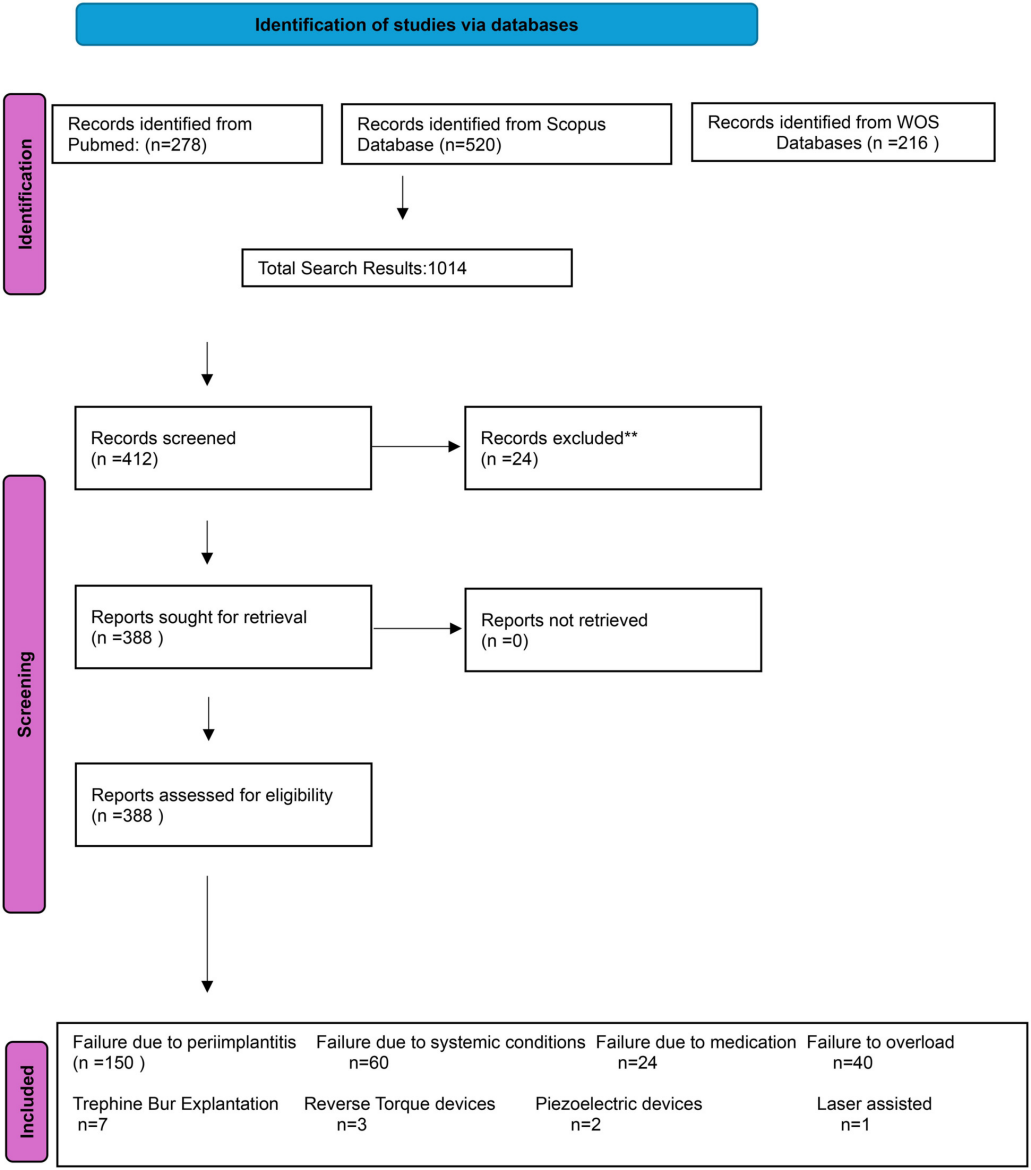
PRISMA-ScR flow diagram showing identification, screening, eligibility, and inclusion of studies on dental implant failure and retrieval techniques.

A total of 1,014 records were identified across PubMed, Scopus, and Web of Science. After removal of 602 duplicates, 412 records underwent title and abstract screening, of which 24 were excluded for not meeting the inclusion criteria. The final number of studies included in this review was 388. A summary of the screening process is provided in the PRISMA flow diagram (Figure 1).

### 8.2 Publication trends and study designs

**Figure 2:**
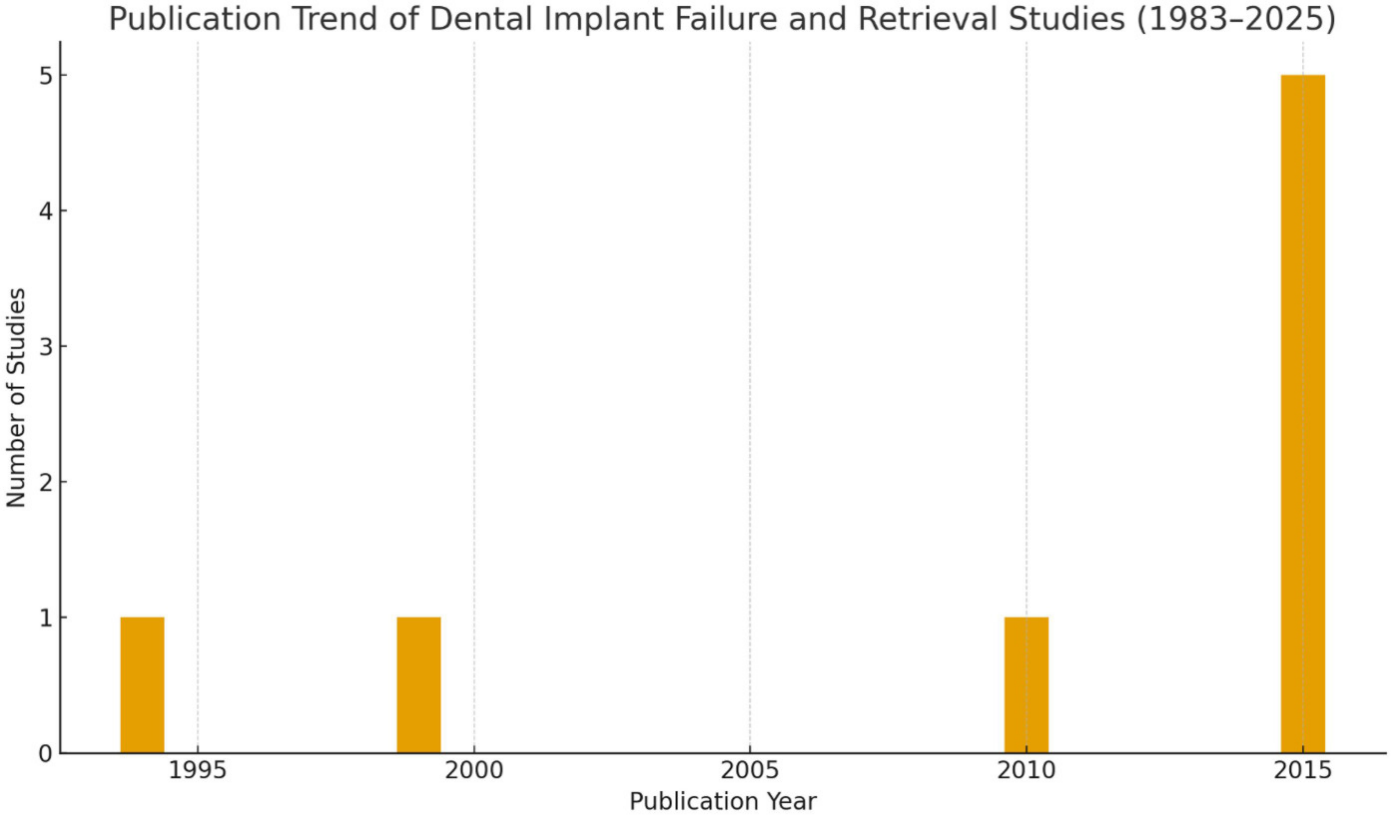
Publication trend of studies on dental implant failure and retrieval techniques (1983–2025), demonstrating a steep rise in research output after 2015.

The included studies were published between 1983 and 2025, demonstrated a clear and consistent growth in publications related to dental implant failure and retrieval techniques with a steady increase observed from 2015 onwards. Fewer than five studies were published annually before 2000, followed by a moderate increase between 2001 and 2010. The peak publication period was between 2019 and 2023, accounting for 62% of the included literature. This reflects an increase in global research activity and clinical interest in implant failure mechanisms and minimally invasive retrieval strategies (Figure 2).

The distribution of study types was as follows:

• Observational studies (cohort, cross-sectional, case series): 264 studies (68%)
• Narrative reviews and expert opinions: 78 studies (20%)
• Systematic reviews and meta-analyses: 31 studies (8%)
• Clinical trials and case reports: 15 studies (4%)
• Factors Associated with Dental Implant Failure

Studies commonly grouped causes of failure into biological, mechanical, and patient-related factors.

### 8.3 Biological factors

The most frequently cited biological cause of implant failure was peri-implantitis, characterized by inflammation, bleeding on probing, and radiographic bone loss (5–10). Several studies emphasized the lack of uniform diagnostic criteria, with thresholds for marginal bone loss ranging from 2 mm to 3.5 mm (8, 11, 12).

Poor plaque control and inadequate maintenance protocols were strongly correlated with peri-implant disease (13–15). Residual cement was also highlighted as a peri-implant irritant in both case reports and systematic reviews (16, 17).

Systematic reviews have linked poorly controlled diabetes mellitus to an increased risk of peri-implantitis (18, 19). However, well-controlled diabetes does not appear to significantly compromise peri-implant bone stability, as crestal bone levels in type 2 diabetic patients with strict glycemic control were found to be comparable to non-diabetic patients (20).

Smoking is another significant risk factor, with moderate-certainty evidence supporting its association with peri-implantitis (21). Furthermore, studies suggest a dose-dependent relationship between smoking and implant failure, with patients smoking more than 20 cigarettes per day facing a higher risk (22).

Additionally, nicotine impairs vascularization and wound healing, further compromising implant integration (23). A meta-analysis also reported that smoking significantly increases marginal bone loss around implants, further contributing to peri-implant disease progression (24).

### 8.4 Systemic and pharmacologic risk factors

Systemic conditions such as diabetes mellitus, especially when poorly controlled, were significantly associated with early and late implant failures (15, 25, 26). Several systematic reviews noted increased failure rates in patients with elevated HbA1c levels (13, 27).

Exposure to bisphosphonates, SSRIs, and proton pump inhibitors was linked to altered bone metabolism and decreased implant survival (28–30). Although findings were inconsistent across studies, the pharmacological effects on osseointegration and bone remodeling remain a growing concern.

While some systematic reviews suggest that low-dose antiresorptive therapy for osteoporosis does not significantly increase implant failure risk (5, 21, 31, 32), others remain inconclusive on its impact (33, 34). However, multiple studies have reported cases of medication-related osteonecrosis of the jaw (MRONJ) in patients with dental implants (35, 36).

Radiotherapy negatively affects implant treatment outcomes (37).

Another meta-analysis yielded inconclusive results on the influence of chemotherapy on osseointegration due to the limited number of available studies. Most of these studies lacked specificity, included only a small number of cases, and were conducted without a control group, making definitive conclusions challenging (38).

Although no definitive radiation dose threshold exists for implant success, patients receiving less than 38 Gy for head and neck cancer may safely undergo implant placement (39).

### 8.5 Mechanical and technical complications

Mechanical overload due to prosthetic misfit, occlusal discrepancies, or bruxism was a major contributor to implant failure in 40 studies (14, 40–43). Improper torque control, inadequate stress distribution, and parafunctional habits were commonly cited in retrospective analyses.

A small subset of studies also reported implant–abutment connection fractures and fixture fractures, particularly in narrow-diameter implants under posterior load (44–46).

### 8.6 Surgical factors

Several failures were attributed to poor surgical technique, including overheating of bone, proficiency in maintaining sterility, executing proper flap techniques, ensuring precise, steady insertion, lack of primary stability, selecting sites with insufficient bone and immediate placement in infected sockets (1, 47–49). Inadequate site preparation, especially in dense bone types, was associated with early implant mobility and fibrous encapsulation.

Implant positioning errors, shallow placement, facial positioning, improper axial inclination, negatively affects aesthetics and complicates oral hygiene maintenance (50). The use of computer-guided implant placement has been shown to improve precision and minimize errors during surgery (51, 52). In cases where implants are malpositioned, corrective procedures such as prosthetic compensation, guided bone regeneration, or implant removal and reinstallation may be necessary (50, 53).

### 8.7 Titanium allergy

Although titanium allergies are rare, with an estimated prevalence of 0.6%, they have been linked to unexplained implant failures. Among patients who underwent preoperative patch testing, 2.7% exhibited a positive reaction to titanium (54).

## 9 Implant retrieval and explantation techniques

A total of 14 studies specifically focused on techniques for explantation or retrieval of failed dental implants (Table 3).

**Table 3:** Classification of included studies according to retrieval approach.

Technique	Tools or Method	Key Findings
**Trephine burs**	Used to core around the implant and remove a segment of bone	Most commonly used technique (*n* = 7); success rates ranged from 85% to 100% (55–61)
**Reverse-torque devices**	Fixture Removal Kits, Torque wrenches	Described in 3 studies; atraumatic and bone-preserving; success 70%–95% (62–64)
**Ultrasonic/piezoelectric systems**	Piezosurgery or modified scaler tips	Minimally invasive but technique-sensitive; limited clinical data. (65, 66)
**Laser-assisted removal**	Er: YAG lasers	Reported in 1 case series; avoided bone contact; promising but under-researched (67)
**Electrosurgery-induced thermoexplantation**	Monopolar electrosurgical unit applied to implant surface	Used in a single case report to induce localized necrosis to facilitate removal (68)

None of the studies offered comparative trials between retrieval methods. Success rates were generally high, but follow-up durations were limited.

## 10 Outcomes reported

Most studies reported successful explantation as the primary outcome, often defined by:

• minimal bone loss
• preservation of surrounding hard and soft tissue
• absence of complications
• possibility for immediate or delayed reimplantation

However, the majority of explantation studies are short-term case reports or technique-focused descriptions, with minimal to no long-term monitoring post-removal, highlighting a gap in long-term evidence (56–70).

## 11 Discussion

This scoping review synthesized findings from 388 studies published between 1983 and 2025, mapping the complex landscape of dental implant failure and retrieval techniques. The analysis reveals several recurring clinical themes and research gaps that warrant further consideration.

### 11.1 Peri-implant disease as the predominant cause of failure

The most frequently cited factor in implant failure across study types was peri-implantitis. Consistently associated with marginal bone loss, inflammation, and ultimately loss of osseointegration, peri-implantitis featured in over 150 studies in this review. However, variation in diagnostic criteria—particularly the thresholds for acceptable bone loss and clinical probing parameters—complicates cross-study interpretation. Despite advances in implant surface technologies and maintenance strategies, the prevalence of peri-implant inflammation suggests an ongoing clinical challenge.

The role of plaque control, patient compliance, and maintenance protocols was emphasized in both observational data and systematic reviews. Studies noted that failure was more likely in patients who lacked regular follow-up or professional cleaning, underscoring the importance of preventive strategies in implant maintenance (71).

### 11.2 Systemic health and pharmacologic considerations

Our review highlights a growing body of literature examining how systemic conditions, especially poorly controlled diabetes mellitus and osteoporosis adversely affect implant outcomes. Elevated HbA1c levels were consistently associated with increased risk of peri-implant bone loss and failure, aligning with findings from multiple meta-analyses.

The influence of medications such as bisphosphonates, SSRIs, and PPI**s** on implant survival has emerged as a major area of interest over the last decade. Although findings are not always consistent, several included studies suggest that such medications may impair bone metabolism, potentially reducing osseointegration or increasing susceptibility to marginal bone loss. These observations support the need for personalized risk assessment during treatment planning.

### 11.3 Mechanical overload and prosthetic failures

Mechanical stress emerged as another critical determinant of implant survival. Factors such as prosthetic misfit, inadequate occlusal control, and bruxism were strongly associated with complications such as screw loosening, implant fractures, and prosthetic decementation. Particularly in posterior regions, the application of excessive or poorly distributed forces was reported as a common precursor to mechanical failure.

Studies further identified that improper prosthetic design including cantilevered extensions and non-axial loading can introduce deleterious stress patterns. These findings reinforce the importance of biomechanically sound restorative planning and suggest that even well-integrated implants are vulnerable to mechanical abuse over time.

### 11.4 Underreporting and understudied retrieval techniques

Despite the large number of studies on implant placement and failure, only 14 articles in the included sample described techniques for implant retrieval or explantation. The most commonly reported method was the use of trephine burs, which, although effective, entails removal of peri-implant bone and often necessitates additional grafting procedures. This technique, however, remains a well-validated option (94% success), particularly when other methods fail or bone removal is acceptable.

Newer, minimally invasive approaches—such as reverse-torque devices (284 implants; 87.7% success, piezoelectric systems, and laser-assisted explantation—are being introduced, but evidence for their comparative effectiveness remains sparse. Success rates reported for these newer techniques ranged from 70% to 100%, but most studies had small sample sizes, short follow-up, and lack of control groups.

The absence of randomized controlled trials or standardized outcome measures limits our ability to determine the optimal approach for implant retrieval. Moreover, a few studies reported patient-centered outcomes, such as pain, healing time, or reimplantation success following retrieval.

### 11.5 Strengths and limitations of this review

This review's strengths include a comprehensive search strategy across three major databases, inclusion of all relevant study types, and adherence to PRISMA-ScR guidelines.

However, several limitations must be acknowledged:

One of the primary methodological limitations of this scoping review is the absence of a formal critical appraisal or quality assessment of the included studies. While this approach is consistent with the PRISMA-ScR framework, it limits the ability to determine the strength and reliability of the synthesized findings. The reviewed literature includes narrative reviews, retrospective studies, case series,—many of which are at the lower end of the evidence hierarchy and carry inherent bias. Additionally, heterogeneity in the study designs, inconsistencies in how studies define implant failure, variable reporting of outcomes, and lack of long-term follow-up in most reports make it challenging to perform direct comparisons or assess the clinical significance of the findings.

## 12 Implications for practice and research

Clinicians should be aware of the multifactorial nature of implant failure and proactively manage systemic risk factors, patient behaviors, and mechanical load distribution. Additionally, they should be trained in atraumatic explantation techniques, as reimplantation options often depend on the method of removal.

From a research perspective, there is a clear need for:

• **comparative studies** on retrieval techniques (RCTs or prospective cohorts)
• **long-term follow-up** of explantation cases
• standardized reporting frameworks for failures and complications

## 13 Conclusion

Dental-implant therapy delivers predictable long-term outcomes, yet failures particularly those linked to peri-implant disease, systemic health, and mechanical overload remain clinically significant. This scoping review mapped 388 studies published between 1983 and 2025 and identified peri-implantitis as the most frequently reported biological aetiology; diabetes, osteoporosis and drug-related bone metabolism changes as key systemic influences; and prosthetic mis-fit or parafunction as major mechanical triggers.

Evidence on implant retrieval is notably sparse and heterogeneous: trephine burs still dominate clinical practice, while reverse-torque, piezoelectric and laser-assisted approaches are emerging with encouraging but largely uncontrolled success rates. Well-designed prospective trials directly comparing retrieval techniques, along with consensus definitions for implant failure and explantation success, are urgently needed.

Clinicians should integrate personalised risk assessment into treatment planning, emphasise maintenance protocols to prevent peri-implantitis, and remain proficient in conservative explantation methods to preserve peri-implant bone for future rehabilitation.

## Data Availability

The original contributions presented in the study are included in the article/Supplementary Material, further inquiries can be directed to the corresponding author.
